# The co-production process of an assessment programme: Between clarifying identity and developing the quality of French-speaking Belgian community health centres

**DOI:** 10.1186/s12961-024-01112-y

**Published:** 2024-02-20

**Authors:** Madeleine Capiau, Jean Macq, Sophie Thunus

**Affiliations:** Institute of Health and Society (IRSS), Université catholique de Louvain, Clos Chapelle-aux-Champs, 30, 1200 Brussels, Belgium

**Keywords:** Assessment programme, Co-production process, Primary care, Community health centres, Multidisciplinary teams, French-speaking Belgium

## Abstract

**Background:**

The assessment of primary care organizations is considered to be essential for improving care. However, the assessments’ acceptability to professionals poses a challenge. Developing assessment programmes in collaboration with the end-users is a strategy that is widely encouraged to make interventions better targeted. By doing so, it can help to prevent resistance and encourage adherence to the assessment. This process, however, is rarely reported. This paper aims to fill this gap by describing the process of the co-production of an assessment programme for community health centres (CHCs) affiliated to the Federation of Community Health Centres (FCHC) in French-speaking Belgium.

**Methods:**

We conducted a documentary study on the co-production of the assessment programme before carrying out semi-structured interviews with the stakeholders involved in its development.

**Results:**

CHCs in French-speaking Belgium are increasing in number and are becoming more diverse. For the FCHC, this growth and diversification pose challenges for the meaning of CHC (an identity challenge) and what beneficiaries can expect in terms of the quality of organizations declaring themselves CHC (a quality challenge). Faced with this double challenge, the FCHC decided to develop an assessment programme, initially called Label, using participatory action research. During the co-production process, this initial programme version was abandoned in favour of a new name “DEQuaP”. This new name embodies new objectives and new design regarding the assessment programme. When studying the co-production process, we attributed these changes to two controversies. The first concerns how much and which type of variety is desired among CHCs part of the FCHC. The second concerns the organization of the FCHC in its capacity as a federation. It shed light on tensions between two professional segments that, in this paper, we called “political professionalism” and “pragmatic professionalism”.

**Conclusions:**

These controversies show the importance of underlying challenges behind the development of an assessment programme for CHCs. This provided information about the evolution of the identity of multidisciplinary organizations in primary care. Issues raised in the development of this assessment programme also show the importance of considering assessment methods that reflect and embody the current realities of these organizations and the way of developing these assessment methods.

## Introduction

Primary care, an essential part of programme in the health system, is under pressure due to current demographic, epidemiological and economic challenges [1, 2]. The quality of primary care has therefore become an area of concern for public authorities [1]. Quality assessment is now a key factor in the regulation of primary care organizations [3]. An increasing number of quality assessment programmes have been introduced, as has been observed for some time in the area of hospital care [4, 5]. Those programmes incorporate different methods, such as assessment by care consumers, and external assessors as well as self-assessment by the professionals themselves [4, 5].

Studies have shown that these assessment programmes can bring about an improvement in the quality of care [6–8]. Other studies have shown, however, that incorporating them into the management of health organizations leads to mixed responses and sometimes conflicting reactions from professionals [6, 9–15]. Such reactions can be explained by the professionals’ differing interests, perceptions and values regarding assessment [13]. Discrepancies can cause competition between professionals, undermining the overall process and the results of the assessment [16].

Involving stakeholders in the development of an assessment programme is one way to anticipate possible resistance [13, 17, 18]. Co-production allows the different stakeholders (those who commission it, those who carry it out and those who are themselves assessed) to work closely together [19]. The underlying assumption is that the involvement of stakeholders in the development of these interventions makes them more acceptable. Being better targeted to the realities of the end-users, avoids having assessment programmes that often fail in terms of feasibility, efficiency and public adherence [20, 21]. However, most of these programmes are imposed externally without any consultation of their end-users [18, 22–30]. Stakeholders often have different visions, expectations and knowledge about the assessment. In practice, the time devoted to co-production gives rise to a series of discussions between stakeholders during which they discuss them. Having such conversations should ensure that multiplicity of perspectives is included in the process of co-production [31, 32].

The literature relating to the development of quality improvement programmes focuses solely on the changes that emerge from co-production [33–40]. The literature, however, also recommends that the entire development process be documented, including the time spent comparing different visions and the multiple resulting negotiations between the stakeholders involved in the programme [25, 28, 33, 35, 41–43]. In the area of primary care, there is, to our knowledge, no research which documents the process of co-producing an assessment programme.

The aim of this paper is to describe the process of co-producing the assessment programme currently known as “Développons ensemble la qualité de nos pratiques” (DEQuaP; in English, “Let us develop the quality of our practice together”) intended for community health centres (CHCs; in French *maisons médicales*) affiliated to the Federation of Community Health Centres in French-speaking Belgium (FCHC; *Fédération des maisons médicales*).

Multidisciplinary practice and a territorial approach are the core components of the primary care model of the CHCs. They are healthcare organizations which develop differently according to the needs, culture and practices of the users’ communities and the geographical, political and organizational landscape in which they exist. This model of primary care organizations aims to provide a more appropriate response to the health and care needs of individuals within their communities, in the context of their daily lives. It aims to provide them with general, lifelong, continuous, comprehensive and integrated support from a team of professionals [44].

Some of these CHCs are affiliated with the FCHC. Created in 1981, the FCHC represents 130 CHCs in Wallonia and Brussels. The objective of the federation is to promote a primary-care-centred model and to support its affiliated CHCs. In practical terms, the FCHC supports its members with discussion forums, organizational tools, training programmes and actions in line with the FCHC’s values and objectives. It also assists in the creation of CHCs by supporting new teams in their development. Finally, it represents the CHCs in the various healthcare policy instances [45].

The number of CHCs is increasing as well as their diversity. Although increasing diversification of CHCs is at first sight seen as a potential for quality improvement, it has also seen as a potential risk for the quality of primary care provision [46]. In our results, we have focused on the way some dimensions of quality of care can be affected by these current challenges in the multidisciplinary primary care landscape. These dimensions refer to accessibility, continuity, efficiency and people-centred care. In a context where CHC is not a protected title, it also represents challenges for the identity of the CHCs affiliated with the FCHC. The FCHC therefore decided to develop an assessment programme, initially called Label, to address quality and identity issues encountered by the FCHC-affiliated CHCs. Using a participatory action research, the initial programme version (as planned by the designers) was abandoned in favour of a new name: DEQuaP. This new name embodies new objectives and new design regarding the assessment programme.

By studying the process of co-producing the assessment programme from Label to DEQuaP, we sought to answer the following research questions: (1) Are there recurring topics of discussion during the co-production of the Label assessment programme which reflect tensions related to assessment? How do the discussions in turn influence the assessment programme’s development from Label to DEQuaP? (2) Does the study of co-production tell us anything about the subject of the assessment, that is, CHCs, and, more generally, about multidisciplinary organizations in primary care? (3) What information does the study of co-production reveal about the assessment of multidisciplinary organizations in primary care?

## Methods

Using a qualitative approach, our research drew on two sources of data: documentary analysis and semi-structured interviews.

### Data collection

First, we conducted documentary research on the co-production of the assessment programme. This first data collection aimed to obtain the story of the development of the assessment programme. A variety of sources were consulted: published and unpublished documents, statements, minutes, activity reports and transcripts of discussions between stakeholders involved in developing the assessment programme.

Second, we carried out nine semi-structured interviews with stakeholders involved in the co-production process: two workers from CHCs involved in the pilot phase (CHC pilot 1 and 2), five designers of the assessment programme working for the FCHC (Designer 1, 2, 3, 4 and 5), and two members of the FCHC (FCHC 1 and 2). Most of the people who had taken an in-depth part in the participatory action research had been interviewed. In addition, recruitment stopped when no additional themes emerged from the analysis. This second data collection aimed to understand their experiences and opinions regarding the assessment programme co-production process in more depth (Table 1). Due to the COVID-19 crisis, the interviews were carried out on Zoom. They were carried out between May and August 2020 and lasted an hour on average. The interviewees were given assurance that their answers would be confidential. The interviews were recorded with the consent of each participant.

**Table 1:** Topic areas covered in qualitative interviews

Topics areas	Objective and description
Development history of the assessment programme	To develop an understanding of the assessment programme that had been developed, which included a description of the problem being addressed, the context in which it was developed and the process of development, including its changes
Participant’s experience in the co-production process of the development of the assessment programme	To ascertain information pertaining to the key elements in the process of co-production, such as stakeholder’s involvement and the decision-making process
Participant’s opinion regarding the assessment programme	To obtain reactions about the initial version of the assessment programme and their positions in favour of and against the assessment programme prototype

To broaden our view about the assessment programme co-production and our knowledge of CHCs, we also carried out interviews with professionals working in CHCs (Workers 1–8) as part of another study (Melting Point). This was a qualitative study on the access and use of primary care by vulnerable people in the Brussels Capital Region, commissioned by the Brussels Health and Social Affairs Observatory and supervised by Sophie Thunus [43].

### Data analysis

We used documents to describe the origins of the prototype of the assessment programme and the stages it went through before the final version was implemented. To understand those changes, we analysed the semi-structured interviews with Nvivo 12 qualitative data management software. We shed light on the contrasting reactions of the stakeholders and their positions in favour of and against the assessment programme prototype. It revealed tensions relating to identity as well as to ideological and organizational changes within the Federation’s CHCs. To interpret the tensions discussed during the co-production process, we used the concept of controversy, which has its origin in the sociology of science and technology [40]. This article employs the term “controversy” to designate collective discussion, carried out using a cross-cutting approach, throughout a process, while drawing on scientific and practical knowledge. Controversies raise moral and ideological considerations that often go beyond the initial subject or, in our case, beyond the methodology [41, 42]. Analysis of controversies, in the context of a particular practical situation therefore, makes it possible to identify the strategic and ideological concerns of the stakeholders involved. The last interviews of the Melting Point study helped in seeking a deeper understanding of the essential elements of the controversies.

### Ethics

Ethical approval was obtained from the Hospital Departmental Ethics Committee of Saint-Luc, Catholic University of Louvain, in Brussels. Moreover, the principal researcher, MC, did not contribute to the participatory action research. Her doctoral research began when the assessment programme in its final version (DEQuaP) had already been implemented in the CHCs in 2020.

## Results

The results are presented in three sections. First, we describe how the Label assessment programme emerged from the CHCs of French-speaking Belgium. We then present two controversies that arose from the discussions on the development of the programme. Finally, we present the final version of the assessment programme, called DEQuaP, that took shape after the participatory action research.

### The creation of the Label assessment programme

The diversity of CHCs in French-speaking Belgium is increasing as they grow in number. Some professionals working in CHCs of the FCHC fear that the quality of multidisciplinary organizations may suffer as diversity increases. As explained below, the assessment programme was initially developed as a way of responding to those concerns.

#### The diversification of community health centres

For several years, the landscape of primary care in French-speaking Belgium has been marked by an increase in multidisciplinary organizations, mostly known as CHCs. Although these organizations all offer multidisciplinary services, the way they provide them increasingly differs from centre to centre. CHCs vary in size, and the teams often incorporate different disciplines. Within teams, the division of work varies. For example, some CHCs delegate certain tasks normally carried out by general physicians, such as monitoring treatment for diabetes or blood thinners, vaccines, screening, etc., to nurses. There are also differences in values and priorities. Some organizations value patient participation, and others health promotion or the welfare of workers, and so on. The approach to social and health objectives varies considerably from one CHC to another. For example, tapering plans for drug users are not offered by all CHCs: some supply substitution products, whereas others do not and instead encourage abstinence. There are two models of payment in CHCs: fee-for-service and the capitation fee programme, whereby the National Institute for Health and Disability Insurance pays the CHCs a fixed contribution every month per registered patient. The CHCs register patients and, through their multidisciplinary teams, provide them with preventive and curative care without a personal financial contribution at the point of contact. CHCs can join federations, of which there are now several, including the FCHC, which is the biggest and oldest federation. Members of the FCHC must abide by a list of criteria that includes values, targets and methods, which are set out in a charter. An increasing number of CHCs, however, are choosing to join other federations or not to join one at all: “*In total, there are 180 structures using a capitation fee, of which 120 are FCHC, plus 60 others. Some of them belong to another federation, which is currently very small but is gaining ground, and then there are many others that are not members of any federation*” (Worker 1).

#### The challenges posed by the increasing diversity of community health centres

One of the initial goals of the assessment programme was to demonstrate to users, health professionals and their networks, as well as to the public authorities, the added value of FCHC-affiliated CHCs: “*The public authorities and other stakeholders in the world outside are asking us what the added value of a community health centre is*” (Designer 4). Although increasing diversification of CHCs is, at first sight, seen as a chance for quality improvement, for some members of the FCHC, which pioneered the development of CHCs in Belgium, it also seen as a potential risk for the quality of primary care provision. More specifically, for some respondents, the quality dimensions and values of certain CHCs (whether or not they are affiliated with the FCHC) are moving away from those that were generally accepted when CHCs were created in the 1970s, such as accessibility, continuity, efficiency and people-centred care: “*In general, these are people who do not subscribe to the charter of community health centres and who therefore do not want to defend what they stand for: social justice, equality, solidarity, accessibility of care, etc*.” (Worker 1). Regarding accessibility, it is often undermined by the failure of some CHCs to offer services with flexible hours: “*What I began to see was that some community health centres arranged things for their own convenience to the detriment of accessibility, for example, stating they were open from 9 a.m. to 5 p.m. with an hour’s lunch break*” (Designer 4). Some professionals fear, therefore, that the personal interest of the service workers in such organizations might take precedence over the interests of the users: “*People focus too much on defending workers’ rights and not enough on what we are doing for the patients and for society in general*” (Worker 2). They also fear that there may be a move away from the capitation fee model, currently considered the ideal method, towards a more liberal, business-oriented approach: “*It’s becoming clear that an increasing number of practices that I would deem more commercial are creeping into the capitation fee programme, in order to create a more financially advantageous model*” (FCHC 1). Such organizations are seen as being based on more liberal values, to the extent that participants consider some practices and ways of operating to no longer be justifiable: “*Some of these new organizations do an excellent job but others are veering off course*” (Worker 1). Some professionals claim that unjustifiable practices have already been observed in structures where financial interest takes precedence. One example is registering patients according to social and health criteria and prioritizing the “least sick” and “least poor”: “*It’s also very easy to cheat with fee-for-service, but how do you do that in the capitation fee model? You select people, you only take on those who aren’t that sick, which means you get a lot of money for doing nothing. You could end up with 6000 patients on your book*s” (Worker 1). All these issues could come into conflict with a patient-centred approach, a sub-dimension of quality of care. With regard to continuity issues, professionals have also observed excessive referrals to secondary care by CHCs working with the capitation fee model, motivated by financial interest: “*If I see someone come in with something a bit complicated, if I want to increase my profit proportionally, I have to reduce my workload. So, I pass them on to secondary care. For example, if I get someone who’s depressed and I want to find out why, that takes a lot longer than prescribing drugs*” (FCHC 1).

Issues of maintaining quality in a situation where centres are increasingly diverse arise in a context in which “*community health centre*” is not a protected title. At the moment, CHCs define any practice working with the capitation fee model as primary care. Another risk flagged by the FCHC workers is confusion about goals and methods in all organizations known as CHCs: “The situation is very muddled since some community health centres have developed independently. Some people use the term "*community health centre’ across the board, in the media, whether or not the term is appropriate*” (Worker 1). The generalized use of this term, irrespective of whether community health centres subscribe to the vision of those affiliated to the FCHC, has led some professionals who are members of the Federation to advertise how their practices differ from those of other groups which use the same name, but are different in several respects: “*The way the name ‘community health centre’ was abused, at least in our opinion, raised a huge number of questions. We need a clear set of guidelines if we don’t want to risk damaging the capitation model and penalizing the centres that are doing things properly*” (FCHC 1).

The second objective of the assessment programme is to identify how the CHCs of the FCHC define themselves internally and how they determine the quality of their care. This objective relates to the FCHC’s control over its members. Despite the increase in membership, its power and knowledge of what members do is diminishing: “*At first there weren’t many [community health centres]; everyone knew everyone and they were all activists to some degree. Now there are 120 centres, some of which we never see, nor do we know what they do*” (CHC pilot 2). This objective has to be seen in the context of a lack of monitoring of the affiliated health centres: “*The FCHC can’t control the community health centres. We don’t have the power to go and inspect them either, or to force them to do anything*” (Worker 1).

#### The initial version of the assessment programme

A prototype of the assessment programme was made in 2013 to obtain funding to develop it. The initial prototype proposed by the FCHC was called Label and was based on a self-assessment method for which the CHC teams were responsible. It was based on indicators that reflected the membership criteria of the FCHC. A form of official and visible recognition (a quality label) was to be given to participating organizations. Initially, the self-assessment method was to be overseen by an external third party and an examining committee responsible for the external assessment procedure, that is, the awarding of the Label, which would be valid for 3 years. The committee would have to be an independent body but work closely with the FCHC. Figure 2 presents an overview of this first version of the assessment programme.

**Fig. 1:**
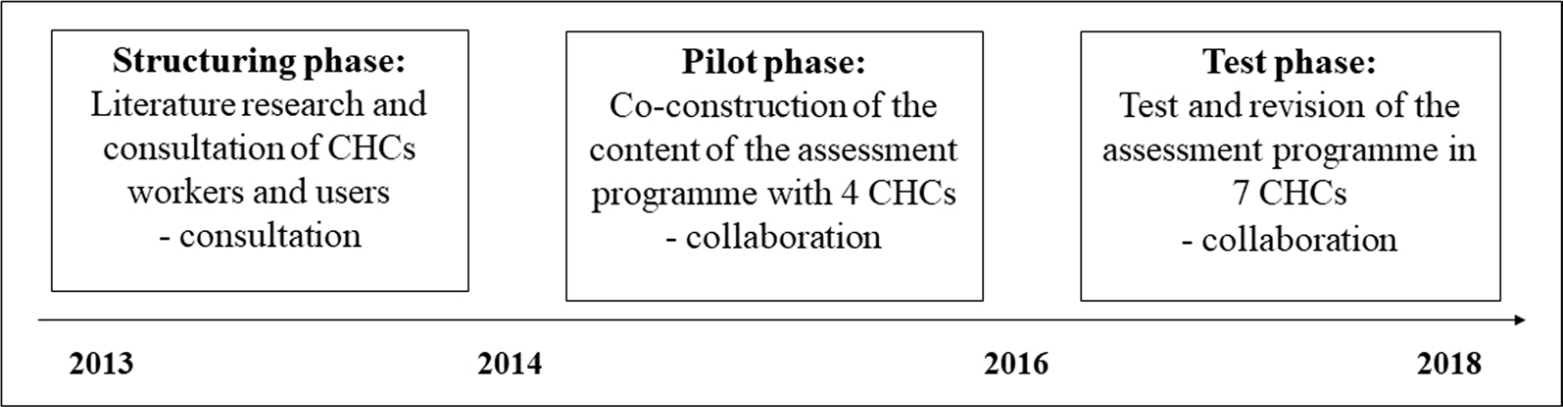
The three phases of the participatory action research of the co-production of the assessment programme

The co-production process aimed to clarify the content, such as quality and identity criteria and dimensions of the CHCs, and the form of the assessment programme by involving CHC professionals and users. The assessment programme co-production process lasted 5 years, from 2013 to 2018, and involved three successive phases, as illustrated in Fig. 1: a phase of literature research and consultation of CHC’s professionals and users, a phase of co-constructing the assessment programme and a phase of testing and revision. Several activities took place during each of the three phases in the development of the assessment programme, with varying degrees of involvement of the professionals, from consultation (phase 1) to collaboration (phases 2 and 3) [47].

### Controversies during the discussions on the development of the assessment programme

The presentation of the prototype of the assessment programme to the professionals of the CHCs in the FCHC gave rise to a series of controversies due to a range of different reactions: “*Everyone took this project as an opportunity to try to promote their own goals. Priorities differ from one person to the next*” (FCHC 1). The controversies arose in two mutually interacting areas: the desired degree of variety in the organizations, practices and forms of commitment of the CHCs affiliated to the FCHC and the division of powers within the FCHC and its missions as the representative body.

#### How much variety should there be in the organizations, practice and advocacy of community health centres?

The discussions about the objectives of the assessment programme revealed an initial controversy regarding the desired degree of variety within the FCHC-affiliated CHCs about organizations, practices and the forms of advocacy the FCHC aspires to. Two conflicting points of view emerged among FCHC professionals: one group was in favour of variety among FCHC-affiliated CHCs and another was in favour of a single standard: “*Some people want there to be a lot of us, to increase the movement’s size and open it up to a greater number of practices, even if they are not entirely on the same course, whereas others prefer a purist approach for the sake of consistency*” (Designer 1).

The position of CHC professionals who favour variety is based on four arguments. First, CHCs need to be able to adapt to the social context of their territory and the needs of care users: “*Our customers are changing, the population is getting poorer and health problems are changing*” (CHC pilot 1). Adaptation to the sociology of a territory, in which the needs of the population are constantly evolving, also requires the professionals working there to diversify their practice: “*We need to be open to forms of practice which are slightly different to those historically developed by community health centres because the world is changing, health professionals are changing, and you need to be open to different practices*” (FCHC 2). According to the same professionals, the FCHC must also open up to forms of advocacy other than those it engaged with when it was first set up because the reasons that professionals work in CHCs have changed in recent years: “*The core main motives of our predecessors and the motives which now lead people to work in community health centres are often very different. Today, there are many young professionals taking jobs in health centres because they like the local project, they like teamwork, but not because they want to change society*” (Worker 2). Finally, the professionals invoke a fourth argument: that variety in CHCs can raise political visibility. The question of how aware public authorities are of the CHCs and the latter’s power to influence health policy depends on the size of the Federation: “*The more of us there are, the greater our political influence; if we end up being a small federation with a handful of community health centres, we will lose all credibility*” (FCHC 2). In light of these arguments, according to these stakeholders, the variety must be reflected in a broadening of FCHC membership criteria: “*Some people are more for openness, saying we need to be open to forms of practice that are a little different to those traditionally followed by community health centres*” (FCHC 2).

On the fringes of the position in favour of variety, which seems to be the majority view, CHC workers deemed to be so-called purists by some professionals (FCHC 2) want to hold onto a strong common identity within the movement. They are calling for greater standardization of the FCHC-affiliated CHCs: “*It’s difficult for the older generation to give up this pure identity*” (Designer 4). These professionals attach importance to continued political activism with respect to the FCHC membership criteria: “*When all the veterans have gone, what then? I don’t want to be pessimistic, but we’ll have lost something all the same. Will things go on as usual? I don’t know*” (Worker 8). According to these professionals, if the commitment to political activism is no longer a key value of the FCHC, there is a risk that no one will defend the CHC model to the public authorities: “*There was a young upcoming generation, 20–25 years old, with their ideas and a message of good news, ready to take over in case the movement lost its way*” (Designer 4). Fears that were raised included a loss of protection for users’ interests, which lay at the heart of the FCHC when it was created and underpinned the commitment of many service providers when the first CHCs were set up in the 1990s: “*[This health care model] sometimes seems to attract young health professionals who want to join community health centres; they recognize their value, but to claim that there is an underlying health model people want to defend, but which not all politicians defend, no one really cares or wants to get involved*” (CHC pilot 2).

The position of the stakeholders regarding the acceptable degree of variety in CHCs had consequences for the way the initial version of the assessment programme, called Label, was perceived. From the point of view of those in favour of diversity, applying the Label assessment programme to CHCs would lead to more stringent FCHC membership criteria and a possible drop in membership to the benefit of other federations, thereby compromising the FCHC in the eyes of the public: “*Is it better to remain a small federation which closes ranks, with a strong restrictive identity, at the risk that all the other centres join different federations?*” (Designer 4). Another risk linked to the initial version of the assessment programme (based on an assessment method common to all CHCs) is that it may penalize adaptation to circumstances or even reduce the ability of the organizations to adjust to the context they find themselves in. Some professionals, therefore, point to a mismatch between a method common to all the CHCs and the many different environments they work in: “*It is not possible to have the same programme in all the community health centres because we’re all different, working in different contexts. We have a huge variety of health centres; no two centres are the same*” (Worker 6). Those stakeholders also fear that the assessment programme could interfere with their professional practices, which are unavoidably linked to the environment they work in and are therefore constantly evolving. From the point of view of those who advocate standardizing the CHC members of the FCHC, the assessment programme is sometimes considered a mainstay, or even a shield against the pressures to diversify the FCHC-affiliated CHCs: “*They saw it [the Label assessment programme] as an opportunity for an overhaul, to force certain health centres to improve their practice*” (Designer 1).

#### What kind of federation do community health centres need?

The approach to assessment proposed by the FCHC for its CHCs also gave rise to a second controversy regarding both the division of power within the FCHC and the missions of the FCHC, which we will study as an organizational controversy.

On the one hand, when analysing the positions regarding the role of the FCHC within the Label assessment programme, we noticed two types of logic with respect to the division of power within the FCHC: a centralized logic and a decentralized one. Some professionals thus favour a federation that takes charge of ensuring that CHCs continue to respect the membership criteria: they call for a centralized division of power. They also saw the assessment programme as an opportunity to confer this role upon the federation: “*There were teams that wanted to use this thing to have a clear-out, to get rid of any teams which they felt weren’t working properly*” (Designer 1). On the other hand, other professionals were in favour of autonomous practices and ways of working, involving complete independence vis à vis the Federation, that is, decentralized power. Those defending this position often felt that the FCHC was too controlling: “*Community health centres have the impression that the FCHC wants to force certain choices upon them, that we want to go and see what they’re up to, to stick our nose into their business*” (Designer 1). Consequently, these professionals strongly opposed the Label assessment programme: “*This was, at least at first, their [the professionals’] experience of the project. They were asking: “What is this FCHC which is sticking its nose into our business and assessing how we work, telling us if we’re doing things right or not?*” (Designer 1). They saw it as a threat to their professional autonomy and believed it demonstrated a lack of consideration and trust on the part of the federation towards its members: “*The community health centres saw us [the FCHC] as the eye of Moscow*” (Designer 2).

The professionals working in CHCs also disagreed on the missions of the FCHC as the representative body. On the one hand, for some professionals, the main objective of the FCHC was to engage in political advocacy by promoting health policy based on a programme organized around primary care: “*Some of them [the health centres] are politically very committed and want that approach to be the main focus of the federation’s work*” (Designer 1). On the other hand, other professionals called for the federation to give more support to the development of multidisciplinary group practice focused on public and community health: “*There are others who believe that the community health centres and their federation should not be politicized at all, in the sense of having a political agend*a” (Designer 1). Other professionals qualified this sentiment by stating that, although it is important to defend the primary health care model, it should no longer be given priority in the federation’s political advocacy: “*I think the FCHC should reflect people’s expectations of our practices. I’d rather have a dialogue about our needs, focusing on the local community*” (Worker 7).

### Version 2.0 of the assessment programme: Label becomes DEQuaP

**Fig. 2:**
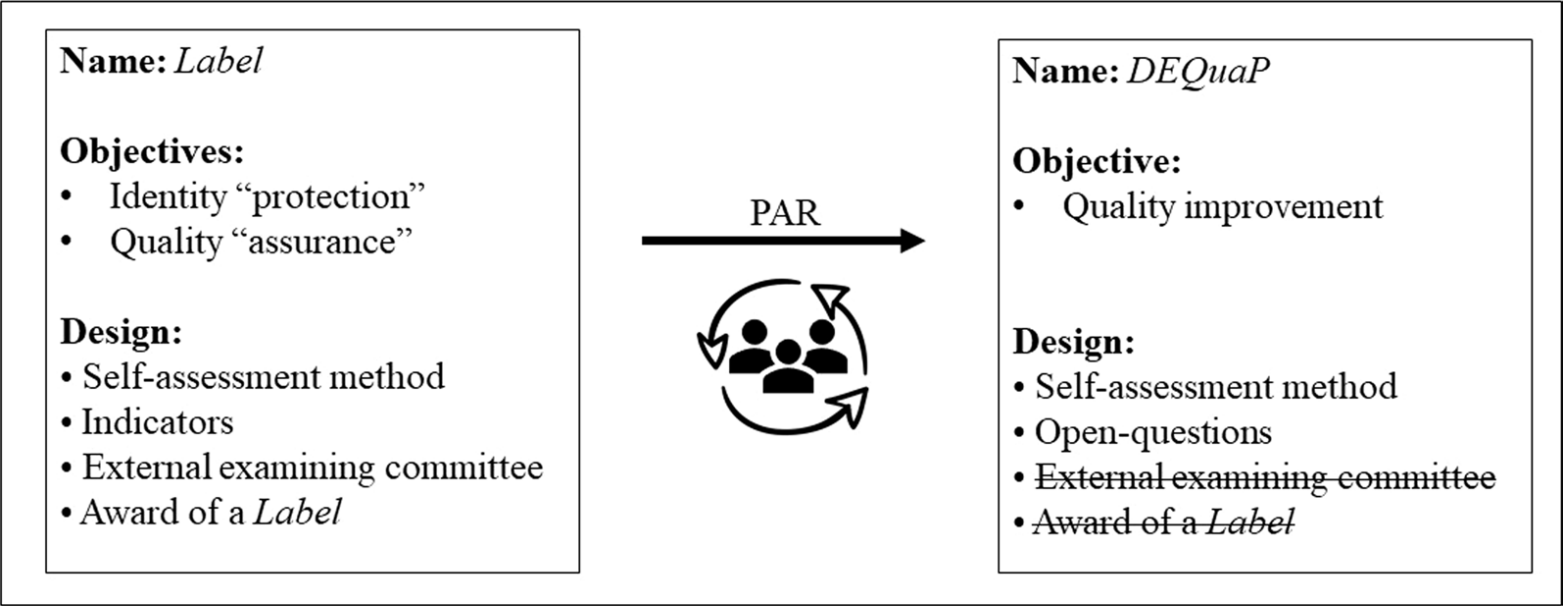
Identity card of the two versions of the assessment programme and evolution of the name, objectives and design through the participatory action research (PAR)

The identity and organizational controversies raised by the development of the assessment programme Label have led to contrasting reactions to it. Having opted for participatory action research to develop the assessment programme, the designers stressed the importance of adapting it to the empirical reality, the reality of the field: “*We opted for a highly participatory methodology in order to ensure ownership by the field, but also to develop a tool and an approach that were really in tune with the needs of the CHCs. This choice proved to be highly relevant*. *This methodology produces rich material and enables workers and patients to get involved concretely and propose orientations that are better suited to the different contexts of CHCs*” (Designer 1). The contrasting discussions and exchanges during the participatory action research thus led the designers to make major changes in the Label assessment programme: “*We changed the direction of the project after the initial investigations: a balance was found between all points of view*” (Designer 3). Changes were made to the objective, name and design of the assessment programme. Figure 2 shows an overview of this second version of the assessment programme and the differences from the first.

Firstly, the designers realized that it was necessary to redefine the objective, at the risk of killing the whole programme. In this view, the “identity objective”, which is protecting the FCHC’s CHCs from the competition resulting from the increasing supply of primary care organizations, was a challenge that went beyond the constraints of the Label assessment programme: “*We wasted time mixing up quality and identity, when in fact the two issues are quite different. Trying to work on both aspects simultaneously was excessive and contradictory. We ran the risk of killing off the desire to improve quality by pasting the Label over the membership criteria*” (Designer 5). The quality improvement objective was therefore selected, and inviting the multidisciplinary teams to reflect on their practices and on the way their CHCs were organized became therefore the only goal of the assessment programme. The focus was placed on enhancing the culture of quality in the CHCs, to demystify the assessment process: “*The strategy we had defined was to make everyone comfortable with this assessment challenge which was not part of the culture*” (Designer 4).

Then, following a change of goal, discussions began about changing the name of the assessment programme to make it more specific and to reduce its ambiguity: “*The name caused confusion and some people wanted it changed to better reflect the purpose of the assessment programm*e” (Designer 4). The designers felt that the new name chosen for the assessment programme, *Développons ensemble la qualité de nos pratiques* (DEQuaP; in English, “Let us develop the quality of our practice together”), better reflected the intention of instigating a culture of formative assessment based on the process and not exclusively on measuring results: “*The term ‘Label’ emphasized something we had abandoned, that is the awarding of a label. We looked for a name which reflected what we were creating*” (Designer 5).

Finally, the initial design of the self-assessment method was reworked to better reflect its objective. On the one hand, the quality indicators were replaced by open questions that the multidisciplinary teams were invited to discuss: “*Teams pointed out that it was more appropriate to use questions rather than statements to be endorsed or criticized. Assertions have a normative character, while questions are more stimulating in terms of dynamics. Moreover, if you place too much emphasis on indicators there is a risk that rigid rules might kill creativity. We must avoid indicators that dictate only one way of doing things. What we propose is completely different – a framework, a way of working*” (Designer 5). On the other hand, the idea of a committee to award the label has been abandoned: “*In another context, we could have come up with a different approach where we would define criteria or even standards to be but we don’t have that kind of relationship with our members. Our emphasis is more on the independence of our centres*” (Designer 5).

## Discussion

The development of the assessment programme initially called Label was planned during the co-production process, which was intended to encourage the involvement of the relevant stakeholders and subsequently their support for assessment [17, 18]. Although the literature highlights the importance of involving the participants in the development of quality improvement programmes, there are very few studies on this phase of co-production. Instead, previous studies have focused on results [33–40].

We chose to concentrate on the process of developing an assessment programme for CHCs and to examine the discussion points it raised. In response to our first research question, we identified discussion points incorporating aspects of identity and organization. These related to the subject of the assessment rather than to the development of the method itself appeared to be recurring. We sorted them into two controversies [41, 42].

The first controversy concerned the degree of variety desired by the group made up of CHCs, the FCHC. This controversy calls the collective identity of the FCHC into question. The points of view reported in our results reveal the two main identities under discussion. Some professionals wanted the CHCs to be open to a variety of organizations, practices and forms of advocacy. They believed that this openness would lead to a broadening of the federation’s membership criteria and an eventual increase in the number of affiliated organizations. Other professionals wanted the CHCs in the FCHC to have a stronger single identity. They believed that an increase in the number of organizations entails a major risk of weakening their visibility on the political stage. Moreover, they saw this “identity hybridization” [45] as likely to dilute their values, such as the activist ideology that characterized the creation of the first CHCs in the 1970s.

The second controversy concerns the organization of the FCHC in its capacity as a federation. This second so-called organizational controversy revolves around two issues: the division of power and the federation’s mission as a representative body. Internally, the desired degrees of (de)centralization of power vis-à-vis its member CHCs lie on a continuum between FCHC control over the members and their total autonomy. Externally, the role conferred upon the FCHC in its public relations is either that of politically defending the ideological model of a community health centre or supporting and developing multidisciplinary primary care practices.

By addressing these controversies, we demonstrated that, during the co-production process, the reasons for developing the method had been progressively overshadowed by the organizational diagnosis of the CHCs and their federation. The first lesson learned from the analysis of this process of co-production is therefore that diagnosis should precede the development of assessment programmes. As the sociology of science and technology and implementation science have shown, it is vital to incorporate the characteristics of the context for which it is intended into the assessment programme [48, 49]. Without an initial diagnosis, there is a risk that the development of the programme will be overshadowed by controversies that create a confused situation, thus affecting its future implementation [50, 51]. In the case that we analysed, the issues were so muddled that the designers of the assessment programme had to change the goals and the design, as well as change its name. The new version incorporated the results of the diagnosis so that the assessment programme was better suited to the reality of the environment in which it was to be implemented.

The organizational diagnosis of the FCHC, which was a by-product of the development of the assessment programme, provided information about the context in which the programme was developed, thus providing an answer to the second research question: Does the study of co-production tell us anything about the subject of the assessment itself, that is, the CHCs, and, more generally, about multidisciplinary primary care organizations?

On the one hand, we showed how assessment means different things to different stakeholders. Our results demonstrate that the objectives of the assessment programme are broader than pure assessment and also include strategic and political goals. In the case studied, we could liken the objectives to the desire to introduce an authorization to practice as a community health centre, in a context of increasing diversity, which threatens the foundations of the original concept. The authorization programme, which has been studied in depth in the sociology of professions [52–54], presupposes the existence of a professional movement whose representatives can present a strong single identity to the relevant authorities [55]. In the case we studied, however, we showed that the identity of the CHCs as part of the FCHC was in dispute, as was the role of their representatives. As a result, they do not have the same vision of what needs to be done to protect it, which may explain the contrasting reactions to the assessment programme.

On the other hand, sorting the points of discussion which arose during the development of the assessment programme into different controversies revealed groups of CHCs based on their individual and collective identities and on the way the FCHC itself was organized. From an analytical point of view, these clusters could prefigure the development of what Bucher and Strauss called “professional segments” to describe the formation of sub-groups within professions based on professionals’ practical, theoretical, and methodological preferences [56].

**Fig. 3:**
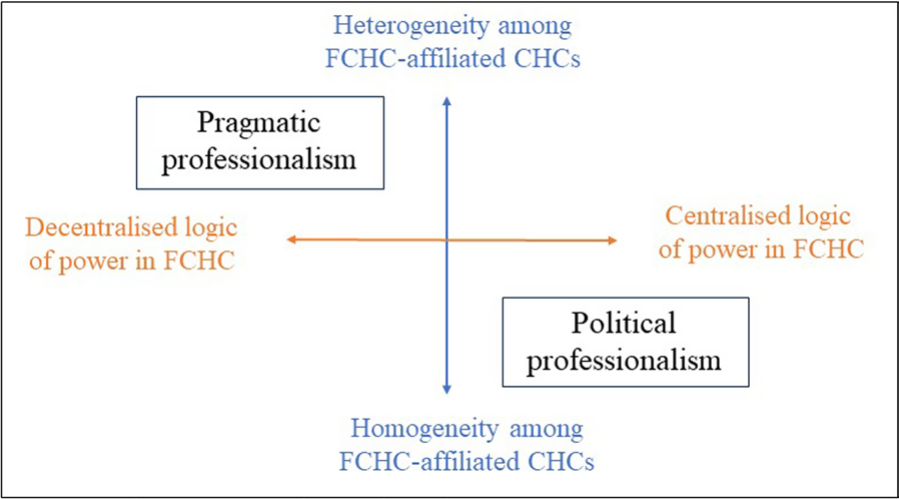
Contours of professional segments called “political professionalism” and “pragmatic professionalism”. On the orange axis: the position relative to the organization of the FCHC; on the blue axis: the position relative to the degree of variety considered desirable within the FCHC

We propose to borrow the concept from these authors to describe the contrasting positions among professionals within the FCHC-affiliated CHCs regarding the identity and central organizing principles of the FCHC. As illustrated in Fig. 3, the controversies reveal the existence of two professional segments that, in this paper, we will call “political professionalism” and “pragmatic professionalism”. Each of these segments used the development of the assessment programme to emphasize its concerns by disputing – sometimes strongly, sometimes less so – the identity and thus the legitimacy of the opposing segment.

The professionals we have associated with the political professionalism segment have at the core of their professional commitment an ideology based on a societal transformation project. That ideology is manifested through activism, philosophy and the organization of work. Consequently, they favour a federation that champions that way of operating by ensuring homogeneity among affiliated CHCs (Fig. 3). This political professionalism can be traced back to the birth of CHCs.

Born in the wake of May 1968, the first CHCs employed professionals who were committed to reforming medicine by replacing the bio-medical, single-practitioner, hospital-centred model with a new social, accessible, local model which would encourage primary multidisciplinary care practices. The professionals who worked in the first such multidisciplinary organizations were therefore general physicians and nurses who wanted to see a thorough reform of medical practice, and who sometimes risked their professional mandates for this political cause [57]. Political activism, fundamental to their struggle from the start, therefore became a principle which the Federation, set up in 1981, applied to all its affiliated health centres and set out explicitly in the membership rules: “*From 1980 onwards, an article in the statutes of the Federation stipulates that the members of the Federation shall commit to a social struggle for health*” [45]. That commitment takes the form of awareness raising.

The professionals associated with the pragmatic professionalism segment see the CHC legacy of advocacy differently. They favour a form of advocacy characterized by a less politicized and more localized professional commitment. They mobilize intermittently around specific issues related to the specificities of their own CHCs [58, 59]. Consequently, their advocacy differs in form and scope from one temporal and geographical context to another, based on the sociological realities of CHCs. This more context-driven approach calls for greater heterogeneity within the FCHC and accepts a greater degree of autonomy for FCHC-affiliated CHCs (Fig. 3). This approach favours a federation that focuses on contextualized realities rather than on an activist role of transforming society.

The power dynamics between these two segments, evident in the controversies that arose during the development of the assessment programme provide valuable insights into the evolution of multidisciplinary practices in primary care. They reflect the current coexistence of political and pragmatic professionalism within those organizations [58].

This coexistence raises real issues, as illustrated in our controversies. Differentiation in the form and extent of activism is sometimes associated by professionals with an abandonment of commitment [58, 59]. More a modification of these forms than an abandonment of commitment, the challenge of this evolution in the landscape of primary care organizations therefore seems to be able to find the right balance between the CHCs’ identities and collective organization. It would simultaneously allow them to cater for the many realities of multidisciplinary primary care organizations and act as a safeguard to avoid the excesses described in our results.

Consequently, this need for balance raised by the challenges of changing forms of professionalism leads us to question how we can conceptualize assessment of multidisciplinary organizations in primary care that takes this into account. The use of standardized quality indicators for all of these multidisciplinary practices in primary care does not fully consider the contextualized and diversified nature of multidisciplinary organizations in primary care; these indicators are regularly criticized for being based on a reductive vision of the realities of the social and health services these centres provide [29, 60–62]. Furthermore, the current highly formalized approach to assessment may lead professionals to understate certain topics, such as their commitment, which reduces the practical relevance of the assessment [40].

In response to our third research question, we encourage the development of assessment methods which offer professionals the opportunity to reflect dynamically on their practices, including their commitment. In this context, other ways of assessing work, such as the inclusion of qualitative arguments (allowing subjective assessments) seem worth exploring when assessing the quality of multidisciplinary organizations in primary care. This does not mean that quantitative indicators should be discarded, but they must not be used exclusively; other considerations, based on listening to professionals, should be considered [63]. We therefore suggest developing and strengthening reflective practices to improve the quality of primary care organizations. These practices offer a twofold opportunity: on the one hand, to give recognition to the pragmatic dimension of professional commitment, care, activities and services and, on the other hand, to offer a means of (re)defining homogeneous integrating principles between professionals in the same organizations and between multidisciplinary primary care organizations. Future research could contribute to demonstrating the added value of qualitative assessment methods in the context of multidisciplinary organizations in primary care and facilitate their development.

Reflecting on the design and content of assessments for the quality of multidisciplinary organizations in primary care also necessitates consideration of how these evaluations are developed. The literature emphasizes professionals’ resistance to quality improvement processes such as assessment, impacting their implementation and outcomes [1–10]. This resistance is often attributed to a lack of consideration for the realities of the field and the professionals who are the future beneficiaries or users of these quality improvement tools [11–20]. Integrating multiple perspectives to bridge the gap between research and users is the focus of the co-production approach [21]. In the development of the assessment programme Label, the designers chose to incorporate these perspectives through participatory action research. Stakeholders, including professionals from CHCs, FCHC workers and CHC’s users, were thus engaged throughout the participatory action research, participating in various activities that facilitated the sharing of views and concerns related to identity and quality issues regarding CHC’s FCHC. Service co-production is becoming increasingly important in healthcare development, and healthcare organizations are expected to involve relevant microsystems in quality improvement interventions. Although this approach is strongly recommended in the field of quality improvement [22], few studies report on what transpired among stakeholders in quality improvement intervention co-production process [11–20]. Given the literature’s reports of negative reactions to quality improvement programmes [1–8], there appears to be a lingering prevalence of tools designed by a top–down approach, without any consultation of future users.

Although co-production processes are experiencing significant growth in the development of healthcare interventions, some of these processes fail. Cooke and Kothari [64] define the co-production approach as tyrannical because it can reinforce the problems it was designed to solve. Power dynamics constitute an overarching and fundamental problem for the outcomes of co-production [65, 66]. Disparities in power commonly exist between scientific experts and other stakeholders, including professionals, as well as citizens. Literature on co-production underscores that elite actors, such as scientists, possess more time and resources and have more knowledge and skills. For all these reasons, they are often considered to be better able to articulate a contribution that is considered relevant and important. In such context, individuals with power can leverage the co-production process to their advantage, shaping the co-production process as they want. This power dynamic impacts the co-production outcome’s quality, utility and legitimacy because those who have power are less likely to result in solutions that resonate with and are usable for non-elite groups [65, 66]. However, in our case study, the outcome of the participatory action research, namely the changes made from Label to DEQuaP, indicates the importance of controversies in reframing the co-production process that finally makes room for recognizing the power and relevance of all stakeholders in the participatory action research. Our results show that the designers of the assessment programme allowed real changes to the way that they had planned the initial version of the assessment programme. The exchange and discussions during the participatory action research encouraged all stakeholders to express themselves, even if they were in opposition to the initial version. Moreover, our case suggests that the culture of the field facilitates such a balanced approach to co-production. Indeed, shared power is inherent to the self-management DNA, participative culture and equity value present within the CHCs of the FCHC. Finally, the adaptations made in the programme studied in this paper suggest that a co-production process based on horizontal rather than hierarchical relationships can help address issues that would could otherwise endanger a whole programme, such as the ambiguity of objectives, which is an important obstacle in the implementation of contemporary, participatory health programmes and policies between all stakeholders implied [66]. In this respect and following Turnhout and colleagues, our paper highlights the importance of shifting from a framework characterized by “power-over” dynamics to a paradigm of “power-with”, where all stakeholders are empowered to generate ideas [66, 67]. In addition, it highlights the importance of contestation and tensions, studied as controversies in our paper, to develop useful interventions [65, 66]. It requires open communication between stakeholders where contestation of interests, differences and potential conflicts can be expressed and considered [65, 66].

## Strengths and limitations

Our paper has some limitations. The assessment programme was developed from 2013 to 2018. Our qualitative interviews were conducted in 2020. There is therefore a risk of memory bias. We also limited ourselves to the French-speaking Belgium FCHC. The scope of the results is therefore limited to the organizations we studied. However, given the transversal nature of the object of study, such as assessment programmes, we believe that the results are relevant to thinking about assessment in other types of organization, in particular primary care and multidisciplinary organizations.

## Conclusion

By studying the co-production process of an assessment programme, this study sheds light on the identity, organization and assessment of community health centres in French-speaking Belgium. Initially designed to develop an assessment programme, the co-production process was subsumed by an organizational diagnosis of these multidisciplinary primary care organizations. This diagnosis highlighted controversies over identity and organization among professionals, which impacted the development of the assessment programme. In light of these controversies, which highlight current professional and societal developments, healthcare organizations should take the time to redefine their identity and structure, and public authorities should support the development of assessment methods that complement existing ones.

## Data Availability

The datasets used and analysed during the current study are available from the corresponding author on reasonable request.

## Abbreviations

CHCs: Community health centres
DEQuaP: Lets us develop the quality of our practices [in French: Développons ensemble la qualité de nos pratiques]
FCHC: Federation of Community Health Centres
